# Genome-Wide Association Studies Reveal Genetic Variation and Candidate Genes of Drought Stress Related Traits in Cotton (*Gossypium hirsutum* L.)

**DOI:** 10.3389/fpls.2018.01276

**Published:** 2018-09-03

**Authors:** Sen Hou, Guozhong Zhu, Yuan Li, Weixi Li, Jie Fu, Erli Niu, Lechen Li, Dayong Zhang, Wangzhen Guo

**Affiliations:** State Key Laboratory of Crop Genetics & Germplasm Enhancement, Hybrid Cotton R & D Engineering Research Center, Ministry of Education, Nanjing Agricultural University, Nanjing, China

**Keywords:** upland cotton, drought stress, genome-wide association study, single nucleotide polymorphism (SNP), RNA-sequencing

## Abstract

Cotton is an important industrial crop worldwide and upland cotton (*Gossypium hirsutum* L.) is most widely cultivated in the world. Due to ever-increasing water deficit, drought stress brings a major threat to cotton production. Thus, it is important to reveal the genetic basis under drought stress and develop drought tolerant cotton cultivars. To address this issue, in present study, 319 upland cotton accessions were genotyped by 55,060 single nucleotide polymorphisms (SNPs) from high-density CottonSNP80K array and phenotyped nine drought tolerance related traits. The two datasets were used to identify quantitative trait nucleotides (QTNs) for the above nine traits using multi-locus random-SNP-effect mixed linear model method. As a result, a total of 20 QTNs distributed on 16 chromosomes were found to be significantly associated with six drought tolerance related traits. Of the 1,326 genes around the 20 QTNs, 205 were induced after drought stress treatment, and 46 were further mapped to Gene ontology (GO) term “response to stress.” Taken genome-wide association study (GWAS) analysis, RNA-seq data and qRT-PCR verification, four genes, *RD2* encoding a response to desiccation 2 protein, *HAT22* encoding a homeobox-leucine zipper protein, *PIP2* encoding a plasma membrane intrinsic protein 2, and *PP2C* encoding a protein phosphatase 2C, were proposed to be potentially important for drought tolerance in cotton. These results will deepen our understanding of the genetic basis of drought stress tolerance in cotton and provide candidate markers to accelerate the development of drought-tolerant cotton cultivars.

## Introduction

Cotton (*Gossypium* spp.) is the most important natural fiber crop and is also a significant oilseed crop. The upland cotton (*Gossypium hirsutum* L.), which accounts for 95% of the annual cotton production worldwide, is the most cultivated species. However, cotton production is limited by various abiotic and biotic stresses. Of them, drought stress becomes a major threat to substantial loss of cotton yield due to the ever-increasing scarcity of water around the world (Pettigrew, 2004). There is a urgent need to ascertain the molecular and genetic basis underlying the cotton response to drought stress and to develop cotton cultivars with improved drought tolerance.

Quantitative trait loci (QTLs) mapping was an effective tool which was generally used to reveal the genetic basis of complex quantitative traits in cotton (Li C. et al., 2013; Fang et al., 2014; Wang et al., 2014). However, using traditional molecular markers, such as restriction fragment length polymorphism (RFLP) and simple sequence repeat (SSR), only a few QTLs related to drought stress were discovered in a wide region (Levi et al., 2009; Saleem et al., 2015; Zheng et al., 2016) because of the narrow genetic diversity and low map density in modern upland cotton accessions (Fang et al., 2017; Huang et al., 2017; Sun et al., 2017). In addition, these QTLs have not been applied to cotton breeding either. Recently, with the development of functional genomics and transcriptomics, a plenty of genes were found to be involved in drought resistance, including protein kinases, transcription factors and some structural genes (Ashraf et al., 2018). And these factors are related to different signal transduction pathway in response to drought stress. For example, *GbMYB5* and *GhWRKY17* are positively involved in response to drought stress (Yan et al., 2014; Chen et al., 2015), while a complete MAP kinase cascade that phosphorylates and activates *GhWRKY59* is involved in abscisic acid (ABA)-independent signaling pathway to regulate cotton drought responses (Li et al., 2017). And overexpression of *GhNAC2* can enhance root growth and improve tolerance to drought in transgenic cotton and *Arabidopsis* (Gunapati et al., 2016). Nevertheless, the functional verification for only few genes related to drought tolerance was reported. How to excavate more drought stress related genes accurately and effectively and to utilize them for breeding drought-tolerant cotton cultivars remain a big challenge.

Compared with traditional molecular markers, single nucleotide polymorphisms (SNPs) are the most abundant DNA variation distributed along the genome, with high density, bi-allelic and co-dominant characteristics. Recent years, the application of SNP arrays (Hulse-Kemp et al., 2015; Cai et al., 2017), sequencing and re-sequencing (Li et al., 2015; Zhang T. et al., 2015) for upland cotton accessions made it possible to improve the resolution of genetic maps and the accuracy of QTL mapping. Genome-wide association study (GWAS) is an effective method, which can associate phenotypes with genotypes in natural populations and reveal vast natural allelic variations and candidate genes based on linkage disequilibrium (LD), and have been widely used in crop plants, such as rice (Huang et al., 2010; Yano et al., 2016), maize (Li H. et al., 2013; Yang et al., 2014), and soybean (Zhang J. et al., 2015). Based on the rapid-developed studies for genome-wide SNPs in cotton, GWAS for several important agronomic traits has been performed. Using CottonSNP63K SNP array and 719 diverse *G. hirsutum* accessions, GWAS was conducted by integrating different environment tests of fiber quality traits with the SNP genotyping data, and forty-six SNPs were found to be significantly associated with five fiber quality traits (Sun et al., 2017). Using the same CottonSNP63K SNP array, 503 *G. hirsutum* accessions were genotyped for a GWAS with sixteen agronomic traits, and a total of 324 SNPs and 160 candidate quantitative trait nucleotide (QTN) regions were found to be significantly associated with these agronomic traits (Huang et al., 2017). With genome-wide resequencing for 318 cotton landraces and modern improved accessions or lines, 119 associated loci, including 71 for yield-related traits, 45 for fiber qualities and three for resistance to *Verticillium* wilt were identified by GWAS (Fang et al., 2017). Similarly, with resequencing 267 cotton accessions, 19 candidate loci for fiber-quality-related traits were reported (Wang et al., 2017). Recently, through integrating genotyping variation and phenotyping data of 13 fiber-related traits across 12 environments for 419 diverse *G. hirsutum* accessions, 7,383 unique SNPs were found to be significantly associated with these traits. The results showed that more associated loci were identified for fiber quality than fiber yield, and fiber genes in the D subgenome were more than those in the A subgenome (Ma et al., 2018). These studies indicated that GWAS was suitable for detecting QTNs of complex traits in plants. Nevertheless, GWAS based on SNP markers and using large natural populations for drought tolerance related traits has not been reported in cotton.

To determine the key QTN regions and candidate genes significantly associated with cotton drought response, we deployed GWAS of drought stress through integrating the genotypic data of upland cotton accessions by the high-density CottonSNP80K array analysis with their various phenotypic data response to drought stress. Further, the candidate genes were screened by integrating GWAS and gene expression data with qRT-PCR confirmation. This study will provide not only elite genetic resources with candidate SNPs but also key genes to accelerate the drought stress improvement of upland cotton.

## Materials and Methods

### Plant Materials

A total of 319 upland cotton accessions, with 306 cultivars/lines collected from China and 13 landraces introduced from the United States, were used in this study. The accessions in China were mainly collected from four different ecological growing regions: the Yellow River region (YRR, 183), the Yangtze River region (YtRR, 82), the Northwestern inland region (NIR, 22), the Northern China region, the specifically early maturation region (NSEMR, 16), and three unknown origin cotton accessions (**Supplementary Table S1**).

### Phenotypic Analysis

In 2015 and 2017, the 319 upland cotton accessions were planted in the green house with hydroponics and in the phytotron with soil culture in Nanjing Agricultural University, Nanjing, Jiangsu Province, China, respectively. Pilot experiments were performed to screen the suitable concentrations of Polyethylene glycol 6000 (PEG 6000) for investigating the drought tolerance capacities of different upland cotton accessions. As a result, 15% PEG 6000 was used as drought stress treatment for water culture and 10% PEG 6000 for soil culture analysis.

All the accessions were grown in 1/2 diluted Hoagland solution (Hoagland and Arnon, 1950). At the stage of seedlings with five leaves, the experimental plants were treated with 15% PEG 6000 as drought stress, and plants only grown in 1/2 diluted Hoagland solution as control. After 48 h, the indicators related to drought tolerance were measured, including plant height (PH), shoot dry matter (SDM), and root dry matter (RDM). At the same time, top second leaf of the plants were sampled to measure proline content (PC), superoxide dismutase activities (SOD), malonaldehyde content (MDA) and soluble sugar content (SS). In addition, seeds from 319 upland cotton accessions were planted in nursery soil with 10% PEG 6000 solution as drought stress treatment and only watering as control. After 7 days, hypocotyl length (HL) and germination percentage (GP) were measured. For water culture experiments, we selected six seedlings with the relatively uniform growth for each treatment. Each two as a biological replicate, and together three biological replicates were set to investigate the traits, including PH, SDM, RDM, PC, SOD, MDA, and SS. For soil culture experiments, we selected 12 well-developed seeds as a biological replicate for each treatment, with three biological replicates to measure HL and GP.

PH was measured by the length from cotyledonary node to top of the plant. SDM and RDM were measured by weight of aboveground and underground part of the plant after drying at 65°C, respectively. PC was determined by acidic ninhydrin reaction as previously described by Bates et al. (1973). SOD activities were determined by measure inhibition of photochemical reduction of nitro blue tetrazolium (NBT) according to the method of Beyer and Fridovich (1987). MDA content was determined by a modified thiobarbituric acid (TBA) reaction (Hodges et al., 1999). SS content was determined by the colorimetric method using anthrone reagent according to Odjegba and Fasidi (2006). HL was measured by the length of hypocotyl. GP was calculated by ratio of germinated seeds number and planted seeds number.

### Factor Analysis

For the drought tolerance evaluation, factor analysis was performed using SPSS software^1^. Kaiser–Meyer–Olkin (KMO) measurement and Bartlett’s statistic were calculated to determine the selected variables. Factors were extracted by the cumulative-contribution-rate-more-than-85% rule and comprehensive evaluation of the drought tolerance based on factor scores (Chen, 2013).

### SNP Genotyping

Genomic DNA of the 319 cotton accessions was extracted according to the method described by Paterson et al. (1993). A CottonSNP80K array containing 77,774 SNPs (Cai et al., 2017), was used to genotype the 319 accessions. Qualified DNA was hybridized to the array following the Illumina protocols. The Illumina iScan array scanner was used to scan arrays, and GenomeStudio Genotyping software (V2011.1, Illumina, Inc.) was employed to cluster SNP alleles and genotyping. All 77,774 SNPs were tested and manually adjusted as described by Cai et al. (2017). The SNP data set with a calling rate < 0.9 and MAF < 0.05 was further filtered, and the high quality data was used for subsequent analysis.

### Population Characteristics and Linkage Disequilibrium Analysis

PLINK V1.90 software^2^ was used to conduct the similarity analysis and clustering of 319 cotton accessions. Based on the distance matrix data (1-IBS, identity-by-state), phylogenetic trees were constructed using TASSEL 5.0 software^3^, and visually edited by Figtree software^4^. The IBS matrix data was used to conduct principal component analysis (PCA). The correlation coefficient (*r*^2^) of alleles was calculated to measure linkage disequilibrium (LD) in each group level using PLINK V1.90, and LD blocks containing SNP loci associated with target traits were generated using the R software package “LD heatmap.”

### GWAS and Identification of Candidate Genes

A total of 55,060 SNPs (calling rate ≥ 0.9 and MAF ≥ 0.05) were used for GWAS. To explore the SNP-trait association, multi-locus random-SNP-effect mixed linear model (mrMLM) (Wang S.B. et al., 2016) was employed using the R package “mrMLM” with the following parameters: Critical *P*-value in rMLM: 0.001; Search radius of candidate gene (Kb): 100; Critical LOD score in mrMLM: 3. And the Q+K model was used. Population structure (Q) matrix was calculated using admixture 1.3 with *k* = 3, and kinship (K) matrix was calculated by the R package “mrMLM”. Putative candidate genes were identified flanking 500 Kb of peak SNPs (the most significant SNPs). Gene ontology (GO) analysis was implemented using AgriGO^5^, and candidate genes in “response to stress” terms were selected for further analysis.

### Transcriptome Sequencing and Quantitative Real-Time PCR Analysis

The seedlings of upland cotton genetic standard line, *G. hirsutum* acc. TM-1, with two simple leaves and one heart-shaped leaf, was treated with 15% PEG. The cotton leaf samples were collected in different time-points after treating 0, 6, 12, 24, 48, and 72 h, respectively. Total RNA was extracted from these samples using the Biospin plant total RNA extraction kit (Bioer Technology Co., Ltd.). After pre-processing the RNA-seq data with an NGS QC toolkit (Patel and Jain, 2012), the reads were mapped to the *G. hirsutum* TM-1 genome using a Tophat spliced aligner with default parameters (Trapnell et al., 2009). The genome-matched reads from each library were assembled with Cufflinks (Trapnell et al., 2012). Cuffmerge was then used to merge the individual transcript assemblies into a single transcript set. Lastly, Cuffdiff was used to detect differentially expressed genes (DEGs) with a cutoff of 0.05 *q*-value. Three biological replicates from each sample were used for all RNA-seq experiments.

For quantitative real-time PCR (qRT-PCR) analysis, first-strand cDNA was synthesized using the reverse transcription polymerase reaction system (Promega, United States) and adjusted to ∼100 ng/μL with a One Drop Spectrophotometer OD-1000+ (OneDrop, Nanjing, China). qRT-PCR was deployed on an ABI 7500 real-time PCR system^6^. The qRT-PCR amplification program previously described by Provenzano and Mocellin (2007) was used. The relative expression level was calculated using the 2^-ΔCT^ method (Livak and Schmittgen, 2001) with three biological and technical replicates, respectively. The expression level of *GhHis3* (Accession No. AF024716) was used as an internal control (Gutierrez et al., 2008). All the primers were summarized in **Supplementary Table S2**.

### Statistical Analysis

Correlation analysis among drought tolerance traits was performed using SPSS software, ^∗^ and ^∗∗^ present the significant differences at the 5% and 1% levels, respectively. qRT-PCR data was analyzed using Excel software and shown as the mean ± SD. Multiple comparison in one-way ANOVA was conducted by LSD method at the 0.05 and 0.01 levels, which were marked by ^∗^ and ^∗∗^, respectively.

## Results

### Phenotypic Variation in Drought Tolerance Related Traits

To evaluate the phenotypic variation under drought stress in the natural population, the seeds or seedlings of 319 upland cotton accessions were treated in PEG stress and in well-watered controls. Nine traits related to drought stress tolerance were measured, including HL and GP at germinating stage; PH, SDM, RDM, PC, SOD activities, MDA content and SS content at seedling stage, respectively. The mean and extreme values of five drought-tolerance traits were lower under drought stress than that in control plants and four traits showed higher values under drought stress condition than that in control plants. The coefficients of variation (CV, %) of nine drought tolerance related traits ranged from 11.77 (HL) to 95.89 (PC) under drought stress, and 11.79 (HL) to 92.70 (PC) under well-watered condition, respectively. Furthermore, it showed higher CV value under drought stress treatment than that in control for most drought-tolerance related traits, indicating the wide variation under drought stress among cotton accessions used in this study (**Table 1**).

**Table 1:** Statistics of various traits related to drought tolerance.

Traits	Control					PEG treatment				
	Minimum	Maximum	Mean	*SD*	CV(%)	Minimum	Maximum	Mean	*SD*	CV (%)
PH (cm)	19.46	38.57	28.11	3.96	14.11	14.47	30.41	23.00	3.32	14.43
SDM (g)	0.20	0.74	0.42	0.10	24.57	0.04	0.49	0.28	0.08	30.06
RDM (g)	0.03	0.19	0.09	0.03	36.52	0.01	0.11	0.05	0.02	40.35
PC (μg/g.FW)	2.96	253.23	42.12	39.04	92.70	14.42	4451.77	833.79	799.55	95.89
SOD (U/g.FW)	14.81	163.95	75.23	27.20	36.15	19.16	635.79	156.96	76.48	48.73
MDA (nmoL/g.FW)	8.68	34.79	15.37	3.82	24.87	10.73	86.80	26.55	11.12	41.87
SS (mg/g.FW)	3.16	22.84	6.30	1.86	29.55	4.89	72.32	20.57	11.78	57.27
HL (cm)	3.07	7.33	5.22	0.62	11.79	2.50	6.08	4.25	0.50	11.77
GP (%)	19.44	100.00	79.07	13.98	17.68	13.89	97.22	73.24	13.99	19.10

*PH, plant height; SDM, shoot dry matter; RDM, root dry matter; PC, proline content; SOD*, *superoxide dismutase activity; MDA, malonaldehyde content; SS, soluble sugar content; HL, hypocotyl length; GP, germination percentage; SD, standard deviations; CV, coefficient of variation; FW, fresh weight.*

The relative values of the nine drought-tolerance traits were further calculated using the ratio of the phenotypic effects value under drought stress and that under well-watered control condition. Relative values of each trait were conformed to Gaussian distribution (**Supplementary Figure S1**). To explore the relationships among nine drought-tolerance traits, correlation analysis was conducted. As a result, relative PH (RPH), relative SDM (RSDM) and relative RDM (RRDM) showed significant and positive correlation each other. We also detected a significant and positive correlation among different biochemical index, involved in relative PC (RPC), relative MDA (RMDA), relative SS (RSS), and relative SOD (RSOD). In addition, relative HL (RHL) showed significant and positive correlation with relative GP (RGP) and RSDM. However, both RHL and RSDM showed a significant and negative correlation with RPC, RMDA, and RSS, respectively (**Table 2**).

**Table 2:** Correlation analysis of drought tolerance traits.

	RPH	RSDM	RRDM	RPC	RSOD	RMDA	RSS	RHL	RGP
RPH	–								
RSDM	0.733^∗∗^	–							
RRDM	0.262^∗∗^	0.203^∗∗^	–						
RPC	-0.063	-0.106^∗^	0.051	–					
RSOD	-0.154^∗∗^	-0.073	0.011	0.242^∗∗^	–				
RMDA	-0.091	-0.111^∗^	0.112^∗^	0.621^∗∗^	0.555^∗∗^	–			
RSS	-0.074	-0.121^∗^	0.002	0.554^∗∗^	0.333^∗∗^	0.596^∗∗^	–		
RHL	0.041	0.142^∗∗^	-0.015	-0.145^∗∗^	-0.030	-0.181^∗∗^	-0.128^∗^	–	
RGP	-0.005	-0.069	0.000	0.024	0.011	-0.007	-0.042	0.127^∗^	–

*RPH, relative plant height; RSDM, relative shoot dry matter; RRDM, relative root dry matter; RPC, relative proline content; RSOD*, *relative superoxide dismutase activity; RMDA, relative malonaldehyde content; RSS, relative soluble sugar content; RHL, relative hypocotyl length; RGP, relative germination percentage. ^∗^ and ^∗∗^*: *the 5% and 1% levels of significance, respectively.*

### Comprehensive Evaluation of Drought Tolerance

In order to identify the drought tolerance of 319 cotton accessions, factor and cluster analyses were performed with relative values of nine drought-tolerance traits. The KMO value was 0.634 (>0.5), and the Bartlett’s statistic value *p* < 0.05, indicating that the raw data was suitable for factor analysis. A six-factor solution that accounted for 89.45% of the total variance was obtained (**Supplementary Table S3**) (Chen, 2013). Factor 1 represented the biochemical index factor at seedling stage, including RPC, RMDA, and RSS. Factor 2 was regarded as the physiological index factor, including RPH and RSDM. Factors 3–6 represented RSOD, RHL, RGP, and RRDM, respectively (**Supplementary Table S4**). In addition, the F factor composite score for the drought-tolerance of each cotton accession was calculated by six F factors. Based on drought tolerance capacity with different F factors, cluster analysis showed that the 319 upland cotton accessions were divided into four groups. Totally, 16, 75, 207, and 21 accessions were clustered into advanced, medium, sensitive and extremely sensitive types to drought stress tolerance with F factor ranging from 0.818 to 1.938, 0.239 to 0.742, -0.637 to 0.209, -1.385 to -0.655, respectively (**Supplementary Table S1**).

### Genetic Variation Based on SNPs

We genotyped 319 upland cotton accessions using the CottonSNP80K array. GenomeStudio software (V2011.1, Illumina, Inc.) was used to genotype with a manual corrected clustering file (Cai et al., 2017). The genotypic data revealed that these cotton accessions possessed a high average call rate of 99.39%. With low-quality (call rate < 90% and minor allele frequency < 0.05) loci filtered, a final set of 55,060 SNPs was obtained, with 30,075 and 24,985 SNPs in the At and Dt subgenomes, respectively. These SNP markers were distributed over the entire genome, expect chromosomes A02, A03, and A04 with less SNP density. In addition, the polymorphism information content (PIC) values ranged from 0.263 to 0.389 among chromosomes, and the mean PIC value of the At and Dt subgenomes was 0.338 and 0.334, respectively (**Table 3**).

**Table 3:** Summary of high quality SNPs by genotyping analysis using CottonSNP80K array.

Chr.	Total SNPs	Filtered SNPs	Chr. size (Mb)	Density of SNP (Kb/SNP)	PIC
A01	3500	2365	99.88	42.23	0.372
A02	1996	1410	83.45	59.18	0.349
A03	2466	1792	100.26	55.95	0.348
A04	1434	1055	62.91	59.63	0.362
A05	3384	2485	92.05	37.04	0.352
A06	4698	2525	103.17	40.86	0.287
A07	3070	2132	78.25	36.7	0.340
A08	7773	4967	103.63	20.86	0.286
A09	3621	2440	75	30.74	0.332
A10	2964	2035	100.87	49.57	0.330
A11	2897	1890	93.32	49.38	0.325
A12	3040	1994	87.48	43.87	0.355
A13	4340	2985	79.96	26.79	0.356
D01	2339	1852	61.46	33.19	0.359
D02	2985	2350	67.28	28.63	0.378
D03	1889	1321	46.69	35.34	0.263
D04	1272	1019	51.45	50.49	0.355
D05	2041	1596	61.93	38.8	0.339
D06	4037	3191	64.29	20.15	0.316
D07	3472	2617	55.31	21.13	0.326
D08	2898	2256	65.89	29.21	0.389
D09	2938	2147	51	23.75	0.296
D10	2130	1701	63.37	37.25	0.338
D11	1866	1409	66.09	46.91	0.335
D12	2562	1911	59.11	30.93	0.324
D13	2162	1615	60.53	37.48	0.322

*Chr, chromosome; PIC*, *polymorphism information content.*

### Population Structure and Linkage Disequilibrium

**FIGURE 1:**
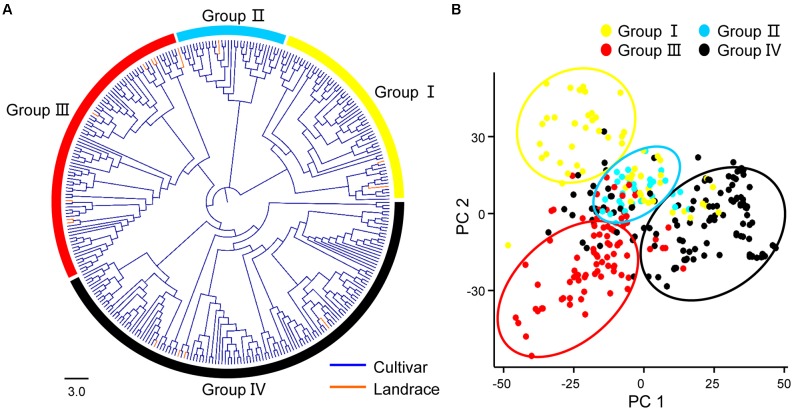
Population structure of 319 upland cotton accessions. **(A)** Neighbor-joining tree of 319 cotton accessions in the panel. Cultivars and Landraces are shown by blue and orange line, respectively. **(B)** PCA plots of the accessions.

**FIGURE 2:**
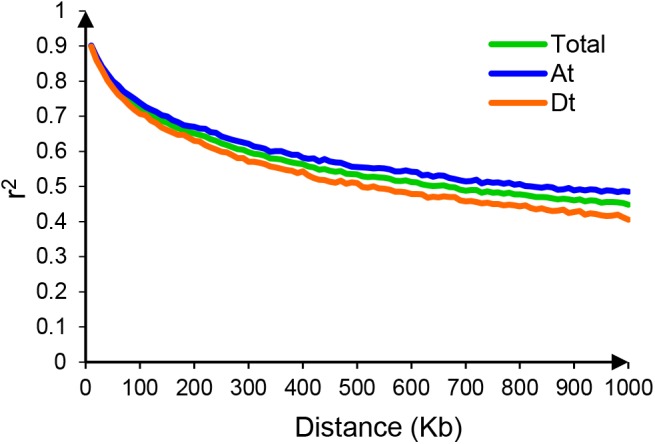
Genome-wide linkage disequilibrium (LD) decay in all cotton accessions. Different colors show the LD decay estimated in different subgenomes.

Principal component analysis and neighbor-joining tree were conducted to infer population stratification. A pairwise distance matrix derived from a modified Euclidean distance for all polymorphic SNPs was calculated to construct neighbor-joining trees using TASSEL 5.0 software. As a result, the 319 accessions could be clustered into four groups, which contained 61, 33, 87, and 138 accessions, respectively. We found that the four clustered groups had no relationships with their geographic origin (**Figure 1A** and **Supplementary Table S1**). Further, clustering data in the phylogenetic tree matched to PCA results well (**Figure 1B**). We also performed linkage disequilibrium (LD) analysis using PLINK software and evaluated Pairwise LD using squared allele frequency correlations (*r*^2^). The LD rate declining to half its maximum value was 980 Kb, with >1000 Kb in At and 760 Kb in Dt subgenome, respectively (**Figure 2**).

### Genome-Wide Association Studies

The GWAS was conducted for the nine traits related to drought tolerance using mrMLM method with Q+K model. Twenty SNPs were found to be significantly associated with six drought tolerance related traits, with both 10 SNPs located on At and Dt subgenome (**Table 4**). In detail, there were three SNPs located on chromosomes A10, D01, and D13 for RRDM, explaining 22.66% of the total phenotypic variation. For RHL, six SNPs were identified on chromosomes A11, A04, A09, D04, D06, and D09, explaining 50.94% of the total phenotypic variation. Two SNPs for RPC were detected on chromosomes A11 and D07, explaining 11.51% of the total phenotypic variation. Two SNPs for RSS were identified on chromosomes D11 and D12, explaining 40.61% of the total phenotypic variation. Four SNPs for RSDM were identified on chromosomes A03, A05, A06, and D06, explaining 29.85% of the total phenotypic variation. For RPH, three SNPs were identified on chromosomes A05, A08, and D12, explaining 26.08% of the total phenotypic variation. The widespread associated loci in different chromosomes indicated that the genetic basis of drought tolerance is complex.

**Table 4:** Summary of SNPs associated with drought tolerance traits.

Traits	SNP IDs	Chr.	Pos. (Mb)	QTN effect	LOD score	*r*^2^ (%)	Number of candidate genes
RRDM	TM36896	A10	100.64	-0.6333	3.41	14.57	59
	TM82142	D13	58.86	-0.5046	3.71	4.82	103
	TM50155	D01	59.85	-0.3164	4.47	3.27	91
RHL	TM37191	A11	6.02	0.0631	4.86	14.14	84
	TM9833	A04	60.55	0.0246	6.21	8.60	83
	TM55926	D04	10.80	0.0192	4.23	4.30	44
	TM30039	A09	2.63	0.0244	3.40	2.76	51
	TM72632	D09	44.33	-0.0398	4.21	16.88	81
	TM62940	D06	60.15	-0.0253	3.71	4.26	55
RPC	TM66782	D07	54.23	-19.9393	4.34	6.35	67
	TM38632	A11	59.82	-17.9695	3.47	5.16	3
RSS	TM77502	D12	3.61	-1.1742	3.89	19.60	69
	TM75380	D11	4.17	1.2065	3.67	21.01	93
RSDM	TM7846	A03	90.78	1.0222	3.56	5.03	33
	TM10434	A05	8.42	1.217	4.92	6.28	130
	TM13658	A06	1.84	0.597	4.73	2.59	76
	TM59389	D06	8.06	-1.1709	3.26	15.95	49
RPH	TM11090	A05	23.64	-0.0826	4.53	5.54	48
	TM29675	A08	96.79	-0.0545	3.26	2.11	63
	TM77685	D12	6.01	0.0905	3.27	18.43	44

*Chr, chromosome; Pos, position; QTN, quantitative trait nucleotide. RRDM, relative root dry matter; RHL, relative hypocotyl length; RPC, relative proline content; RSS, relative soluble sugar content; RSDM, relative shoot dry matter; RPH, relative plant height.*

### Candidate Genes Associated With Significant SNPs Region

**FIGURE 3:**
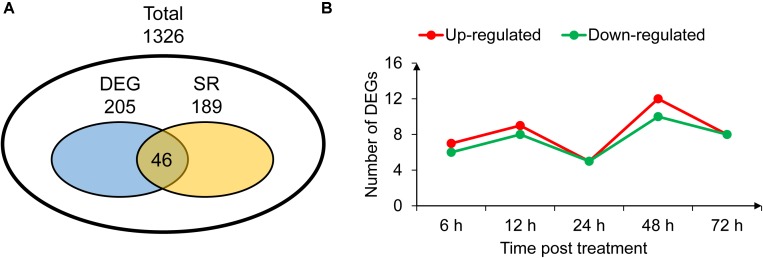
Transcriptome analysis and identification of elite alleles. **(A)** Distribution of the differentially expressed genes (DEGs) and response to stress genes (SR). **(B)** Statistics of the up-regulated and down-regulated DEGs annotated as response to stress.

Candidate genes involved in the 20 significant SNP loci were further mined by referring the LD value in the study. With flanking 500 kb of the significantly associated SNPs and *G. hirsutum* TM-1 genome (Zhang T. et al., 2015) as reference, 1,326 candidate genes with 630 in At and 696 in Dt subgenome were identified. The number of candidate genes associated with six traits was predicted. We found 398 candidate genes from significant SNP regions associated with RHL, while only 70 candidate genes with RPC (**Table 4**). These results implied that HL was involved in a complex process regulated by more regulators while the regulation of PC was relatively specific. GO analysis indicated that 1,226 candidate genes could be mapped to GO background in *G. hirsutum*, which were involved in several biological processes significantly associated with drought stress, such as root system development, regulation of transport, water transport and response to stress. Further, we focused on the GO term “response to stress” (SR), which contained 189 candidate genes (**Figure 3A**). Of them, many genes had been reported to play important roles in drought tolerance, such as *RD2*, *PIP2*, *PP2C*, and *LEA* (Aroca et al., 2006; Samota et al., 2017; Baek et al., 2018; Magwanga et al., 2018), and some key transcription factors, *WRKY*, *NAC*, and *MYB* (Wei et al., 2017; Kiranmai et al., 2018; Negi et al., 2018).

### Transcriptome Analysis and Identification of Elite Alleles

RNA-seq analysis was performed to further explore elite alleles which contributed to drought tolerance. RNA samples were collected from leaves of *G. hirsutum* acc. TM-1 at 0, 6, 12, 24, 48, and 72 h post treatment of 15% PEG. In total, 18 separate libraries were generated with three biological replicates of each sample. The reads generated by the Illumina Hiseq2000 were initially processed to remove adapter sequences and low-quality bases. Approximately 0.82 billion valid reads, each 150 nucleotides long, and roughly 40.8 Gb of nucleotides were obtained. We investigated the expression level of all 1,326 candidate genes [log_2_(RPKM+1) > 1] from GWAS analysis, and found that 205 were differential expression genes (DEGs) with significant induced expression under drought stress condition compared with untreated control. Among these DEGs, 46 were annotated to “response to stress” in GO dataset and other 159 were novel candidate genes (**Figure 3A** and **Supplementary Table S5**). For these 46 DEGs, up-regulated genes are more than down-regulated genes after stress-treated time points (**Figure 3B**). Some up-regulated genes were positively related to stress tolerance such as *RD2* (Samota et al., 2017), and a few down-regulated gene were reported to be negatively related to stress tolerance such as *PIP2* (Macho et al., 2018). In addition, other 143 candidate genes involved in GO term “response to stress” were not induced by drought stress (**Figure 3A** and **Supplementary Figure S2**).

**FIGURE 4:**
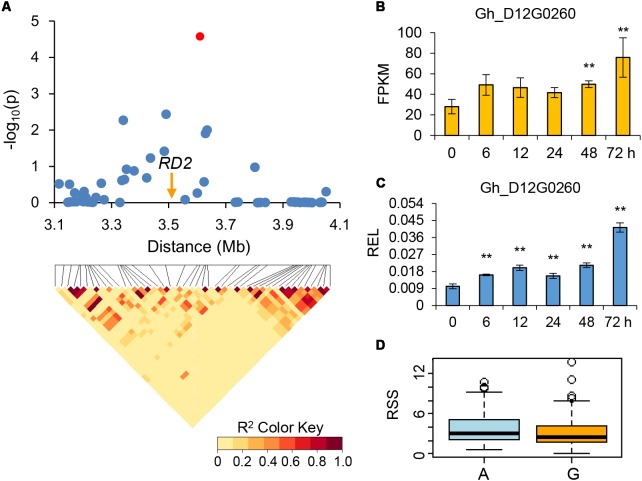
Genome-wide association study (GWAS) for drought-tolerance and identification of the candidate gene *RD2* on chromosome D12. **(A)** Local Manhattan plot (top) and LD heat map (bottom). The red dot indicates the SNP related to the drought-tolerance trait. The arrow indicates the location of *RD2*. **(B)** The expression level of the candidate gene *RD2* calculated via RNA-Seq. **(C)** The relative expression level (REL) of the candidate gene *RD2* calculated via qRT-PCR. **(D)** Differences of relative soluble sugar content (RSS) among two haplotypes. ^∗∗^ means the 1% level of significance.

**FIGURE 5:**
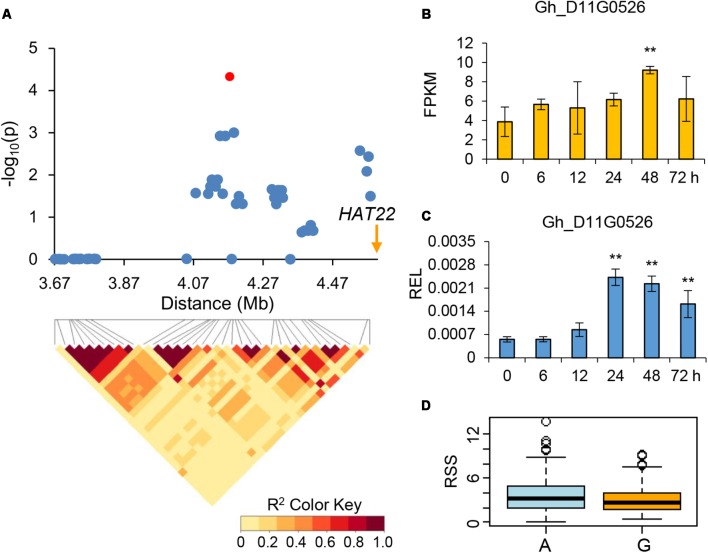
Genome-wide association study for drought-tolerance and identification of the candidate gene *HAT22* on chromosome D11. **(A)** Local Manhattan plot (top) and LD heat map (bottom). The red dot indicates the SNP related to the drought-tolerance trait. The arrow indicates the location of *HAT22*. **(B)** The expression level of the candidate gene *HAT22* calculated via RNA-Seq. **(C)** The relative expression level (REL) of the candidate gene *HAT22* calculated via qRT-PCR. **(D)** Differences of relative soluble sugar content (RSS) among two haplotypes. ^∗∗^ means the 1% level of significance.

**FIGURE 6:**
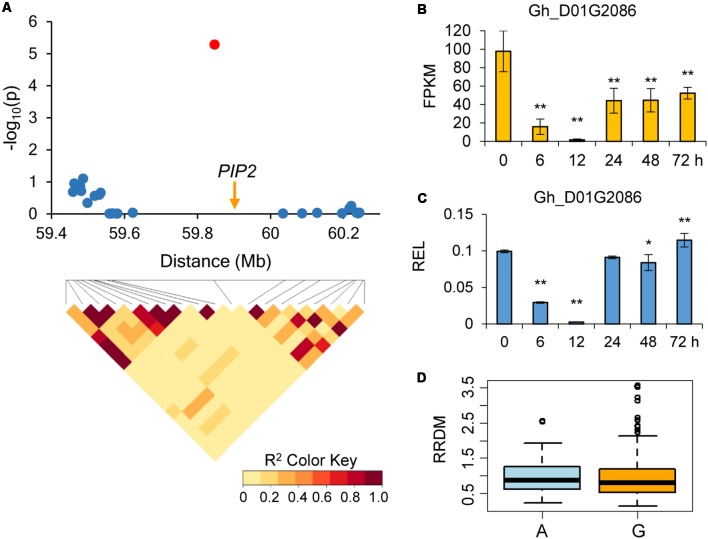
Genome-wide association study for drought-tolerance and identification of the candidate gene *PIP2* on chromosome D01. **(A)** Local Manhattan plot (top) and LD heat map (bottom). The red dot indicates the SNP related to the drought-tolerance trait. The arrow indicates the location of *PIP2*. **(B)** The expression level of the candidate gene *PIP2* calculated via RNA-Seq. **(C)** The relative expression level (REL) of the candidate gene *PIP2* calculated via qRT-PCR. **(D)** Differences of relative root dry matter (RRDM) among two haplotypes. ^∗^ and ^∗∗^ mean the 5% and 1% levels of significance, respectively.

**FIGURE 7:**
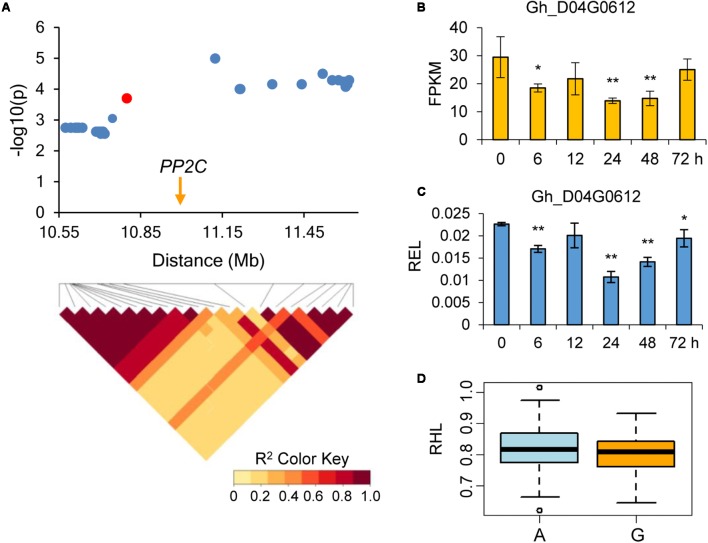
Genome-wide association study for drought-tolerance and identification of the candidate gene *PP2C* on chromosome D04. **(A)** Local Manhattan plot (top) and LD heat map (bottom). The red dot indicates the SNP related to the drought-tolerance trait. The arrow indicates the location of *PP2C*. **(B)** The expression level of the candidate gene *PP2C* calculated via RNA-Seq. **(C)** The relative expression level (REL) of the candidate gene *PP2C* calculated via qRT-PCR. **(D)** Differences of relative hypocotyl length (RHL) among two haplotypes. ^∗^ and ^∗∗^ mean the 5% and 1% levels of significance, respectively.

We combined the GWAS and drought-induced RNA-seq data to explore elite alleles involved in drought tolerance. As a result, four genes were further identified for potential drought tolerance. Within the association signal at D12: 3609663 which explaining approximately 19.60% of the phenotypic variation of RSS, we identified 69 candidate genes. The RNA-seq data showed that one of these genes, which encoded a response to desiccation 2 protein and named as *RD2* (Gh_D12G0260), was continuously up-regulated in all five time points, especially in 72 h post PEG treatment (**Figure 4**). Another gene within association signal at D11: 4173831, which explaining approximately 21.01% of the phenotypic variation of RSS, was also continuously up-regulated after PEG treatment. This gene encodes a homeobox-leucine zipper protein, named *HAT22* (Gh_D11G0526), which has been reported to be related to plant stress tolerance (Liu et al., 2016) (**Figure 5**). Within the association signal at D01: 59846909, which explaining approximately 3.27% of the phenotypic variation of RRDM, we identified a gene down-regulated after drought stress, encoding a plasma membrane intrinsic protein 2 (*PIP2*, Gh_D01G2086), which was involved in root water uptake and tissue hydraulic conductance (Macho et al., 2018) (**Figure 6**). Another gene within association signal at D04: 10799426, explaining approximately 4.30% of the phenotypic variation of RHL, was also down-regulated after PEG treatment. This gene encodes a protein phosphatase 2C, named *PP2C* (Gh_D04G0612), which negatively regulate ABA signaling and stress responses (Baek et al., 2018) (**Figure 7**). In order to validate the reliability of the RNA-seq data, we conducted qPCR assay and confirmed their expression patterns, which were kept consistent in all four candidate genes (**Figures 4**–**7**).

## Discussion

Drought is a serious global problem restricting agricultural development. Previous studies on cotton drought-tolerance mainly pay attention to a single period of cotton development or a small number of indexes or accessions (Chen et al., 2013; Zheng et al., 2014; Ranjan and Sawant, 2015). In this study, 319 upland cotton accessions were collected with a high geographical diversity for genome-wide association studies. A total of nine traits related to drought-tolerance were measured containing two traits at the germinating stage (HL and GP), and seven traits at the seedling stages (PH, SDM, RDM, PC, SOD, MDA, and SS). Proline content has the largest CV, which means that proline content is sensitive to drought stress, and can be regarded as one of the most important trait related to drought tolerance. When suffered drought stress, plants can adapt in three ways: physiological responses such as reducing growth rates, molecular responses such as the increased expression in ABA biosynthetic genes, and biochemical responses such as accumulation of stress metabolites like proline, glutathione, glycinebetaine, polyamines and so on (Fang and Xiong, 2015). Accumulation of proline content, which can increase plant cell’s osmotic pressure and retard plant losing water, is an efficient mechanism to improve drought tolerance. Hypocotyl length associated to the most of SNPs and genes (**Table 4**), indicates that germinating stage is one of the most sensitive stages of cotton, just like sesame (Li et al., 2018). Based on drought tolerance capacity with different F factors, cluster analysis grouped the 319 upland cotton accessions as four types: 16 advanced drought-tolerant accessions, 75 medium drought-tolerant accessions, 207 drought-sensitive accessions and 21 extremly drought-sensitive accessions. In our previous report for salt-tolerance of 304 upland cotton accessions, we detected that 43 accessions were advanced salt-tolerance, 114 medium salt-tolerance, and 147 salt-sensitive (Du et al., 2016). Compared to the salt tolerance, there were relatively few drought-tolerant accessions and need to be further improved for drought tolerance in cotton breeding.

On the basis of the phylogenetic and PCA analysis, we classified the 319 accessions into four groups. However, it showed no obvious relationship with their geographic origin and this result was consistent with most previous study in Upland cotton (Cai et al., 2017; Sun et al., 2017). LD analysis of upland cotton in this study showed that the LD rate declining to half its maximum value was 980 Kb, it is longer than most other crops, such as rice (167 Kb) (Huang et al., 2012) and soybean (420 Kb) (Zhou et al., 2015). The speed of LD decay determines the capacity and resolution of marker-trait association mapping, and the causes of LD mainly including mutation, population bottlenecks, founder effects, drift, selection, migration and population admixture (Morrell et al., 2005; Mackay and Powell, 2007). We speculated that the short history and relatively high rate of self-fertilization of upland cotton breeding in China led to the slower LD decay, implying the more narrow genetic diversity of upland cotton accessions.

Drought tolerance is a complex trait and is regulated by polygenes with small effect. Common GWAS methods are all based on a fixed-SNP-effect mixed linear model (MLM) and single marker analysis, which require Bonferroni correction for multiple tests. When the number of markers is extremely large, the test is too strict. In cotton, combined the resequenced SNP data and phenotyping variation data, GWAS was performed using EMMAX method and the significance threshold was estimated as approximately *P* = 10^-6^. As a result, three loci associated with resistance to *Verticillium wilt* were identified (Fang et al., 2017), implying that mixed linear model with single marker analysis is too strict in GWAS for complex trait such as disease resistance. Multi-locus mixed linear model compresses the markers by the rMLM method and used the selected SNPs to further associate with traits by mrMLM method. Also due to the multi-locus nature, no multiple test correction is needed. So it shows the good effect for complex traits. Here, we used mrMLM of Wang S.B. et al. (2016) and the CottonSNP80K array, genome-wide association studies of drought-tolerance traits with natural population of upland cotton accessions were conducted, and 20 QTNs for drought-tolerance traits were identified. These associated loci were widely distributed across the entire genome and the candidate genes around the loci involved in many biological process, such as root system development, regulation of transport, water transport and response to stress. It demonstrates that drought stress response is controlled by multiple loci and numerous genes.

Compared to yield and fiber quality traits of cotton (Fang et al., 2017; Sun et al., 2017; Ma et al., 2018), the number of reported loci associated with drought tolerance is much fewer. Previous studies have identified several drought-tolerance QTLs in cotton. Based on the progenies from the cross of *G. hirsutum* cv. Siv’on and *G. barbadense* cv. F-177, a total of 33 QTLs were identified under water-limited environments, including five QTLs for different physiological traits, 11 for plant productivity and 17 for fiber quality, respectively (Saranga et al., 2001). In another study, a vast number of QTLs from 42 different studies were surveyed by comprehensive meta QTL analysis, including 132 QTLs for fiber strength, 26 for boll weight, six for gossypol, four for fruiting banch number, five for osmotic potential, three for chlorophyll and so on (Said et al., 2013). However, compared to the GWAS analysis based on high density SNPs, the low density markers and the wide QTL regions showed limitation in identification and utilization of elite genes, especially for marker-assisted selection (MAS) breeding (Zhou et al., 2015). The present study makes progresses in revealing loci related to drought-tolerance traits and identifying SNP loci and candidate genes for drought tolerance. In other studies, using 240 maize accessions and high-density markers, 61 significant SNPs related to drought tolerance were detected by GWAS analysis (Thirunavukkarasu et al., 2014). In rice, through integrating 175 upland rice accessions with 150,325 SNPs, 13 SNP markers and 50 genes associated with yield under drought conditions were identified, further, 10 genes related to drought and abiotic stress tolerance were verified (Pantalião et al., 2016). These studies could be exploited to discover drought tolerance mechanism and contribute to breeding the drought tolerance varieties in crops.

Genetic basis of drought tolerance is complex. Previous studies have reported many genes that are responsive to drought stress in many plants (Song X. et al., 2016; Wang N. et al., 2016; Ma et al., 2017). However, it is difficult to identify candidate genes from the enormous gene pool. In present study, we performed GWAS to identify elite QTNs in natural population and further screen candidate genes by combining with RNA-seq data. As a result, 46 candidate genes with both annotated as response to stress and differential expression under drought stress were selected. Of these genes, *RD2*, encoding a response to desiccation protein, was a key gene for cotton drought tolerance. In rice, expression analyses showed that both *RD1* and *RD2* genes up-regulated under drought stress due to seed-priming, and *RD2* was increased more significantly than *RD1* in tolerance to drought stress, especially on priming with paclobutrazol in drought-tolerant plant and with salicylic acid in drought-sensitive plant (Samota et al., 2017). Another candidate gene *HAT22*, which also named *ABIG1* and encoded a homeobox protein, is a member of the HD-Zip II family. Expression of *HAT22* mRNA increased under drought and ABA treatment. There was less leaf yellowing in *HAT22* mutants than wild type plants with drought conditions. Moreover, some stress related genes such as ABA and ethylene response loci were regulated by *HAT22* (Liu et al., 2016). In the plasma membrane, PIPs are the most plentiful aquaporins with two types, PIP1 and PIP2. There are five and eight isoforms of PIP1 and PIP2 in *Arabidopsis thaliana*, respectively. Generally, PIP1 proteins behave a low efficiency for water transport while PIP2 proteins in plant have a high capacity for water transport (Maurel et al., 2008). Previous studies showed that PP2Cs played a crucial role in regulation of signal transduction pathways. PP2Cs are negative regulators of stress-induced MAPK pathways, ABA signaling and receptor kinase signaling. In turn, expression of *PP2C* was transcriptionally controlled by developmental signals, ABA and stress response (Schweighofer et al., 2004). Abscisic acid (ABA) is an important plant hormone, and regulates plant development and resistance to biotic and abiotic stresses. It controls transpirational water loss by regulating the stomatal opening and closure to resist drought stress. Moreover, ABA can increase plant cell’s osmotic pressure, and expedite chlorophyll breakdown and leaf senescence (Wilkinson and Davies, 2002). By detecting the 46 candidate genes, we found most of them involved in ABA signal pathway and were reported to be related to drought response, such as *PIP2*, *HK1*, *GOLS1*, and *ADC2* (Zhai et al., 2010; Héricourt et al., 2016; Song C. et al., 2016; Macho et al., 2018), indicating ABA signal pathway play crucial roles in response to drought tolerance in cotton. In summary, identification of more drought-tolerance related genes/QTLs enlarges new insight into mechanisms of drought response, and high-throughput genotyping platforms are powerful tools for complex traits dissection and development of drought tolerance varieties in future cotton breeding.

## Conclusion

We genotyped 319 upland cotton accessions using the CottonSNP80K array and phenotyped nine drought-tolerance related traits. By SNP-trait GWAS, we identified 20 SNPs significantly associated with drought-tolerance traits. Integrating the GWAS and RNA-seq data with qRT-PCR verification, we identified four candidate genes *RD2*, *HAT22*, *PIP2*, and *PP2C* for improving drought stress. Our study provides valuable information to explore molecular mechanisms underlying cotton drought tolerance, and supplies new resource for the improvement of drought-tolerance in future cotton breeding efforts.

## Author Contributions

WG conceived the study. SH and YL contributed to phenotyping investigation. GZ and WL performed the GWAS and RNA-Seq analysis. SH, JF, EN, LL, and DZ contributed to the experimental data analysis and discussion. SH, GZ, DZ, and WG wrote the manuscript. All authors read and approved the manuscript.

## Conflict of Interest Statement

The authors declare that the research was conducted in the absence of any commercial or financial relationships that could be construed as a potential conflict of interest. The reviewer ZL and handling Editor declared their shared affiliation at time of review.

## Footnotes

1 http://www.spss.com.cn/

2 http://www.cog-genomics.org/plink2/

3 https://tassel.bitbucket.io/

4 http://tree.bio.ed.ac.uk/

5 http://bioinfo.cau.edu.cn/agriGO/analysis.php

6 http://www.lifetechnologies.com

## Supplementary Material

FIGURE S1 Phenotypic distributions for drought-tolerance traits. RPH, relative plant height; RSDM, relative shoot dry matter; RRDM, relative root dry matter; RPC, relative proline content; RSOD, relative superoxide dismutase activity; RMDA, relative malonaldehyde content; RSS, relative soluble sugar content; RHL, relative hypocotyl length; RGP, relative germination percentage. Gaussian curve is shown in orange line for each trait.

FIGURE S2 Expression pattern of the candidate genes involved in drought response. (A) Genes were annotated as stress response by Go analysis and also differential expression under drought stress. (B) Genes were not annotated as stress response by Go analysis but differential expression under drought stress. (C) Genes were annotated as stress response but not induced expression under drought stress. The scales represent the fold change value of gene expression level.

TABLE S1 Information on 319 cotton accessions used in this study.

TABLE S2 Primers used in this study.

TABLE S3 Eigenvalues of six principal components and their contribution and accumulative contribution.

TABLE S4 Loading matrix of each in six principal components.

TABLE S5 Information on 46 candidate genes related to stress response with significantly differential expression under drought stress.
