# Supplementary figures and images for: Genome-Wide Association Studies Reveal Genetic Variation and Candidate Genes of Drought Stress Related Traits in Cotton (Gossypium hirsutum L.)
Source: Front Plant Sci. 2018 Sep 3;9:1276. doi: 10.3389/fpls.2018.01276 (PMC6129771; doi:10.3389/fpls.2018.01276)

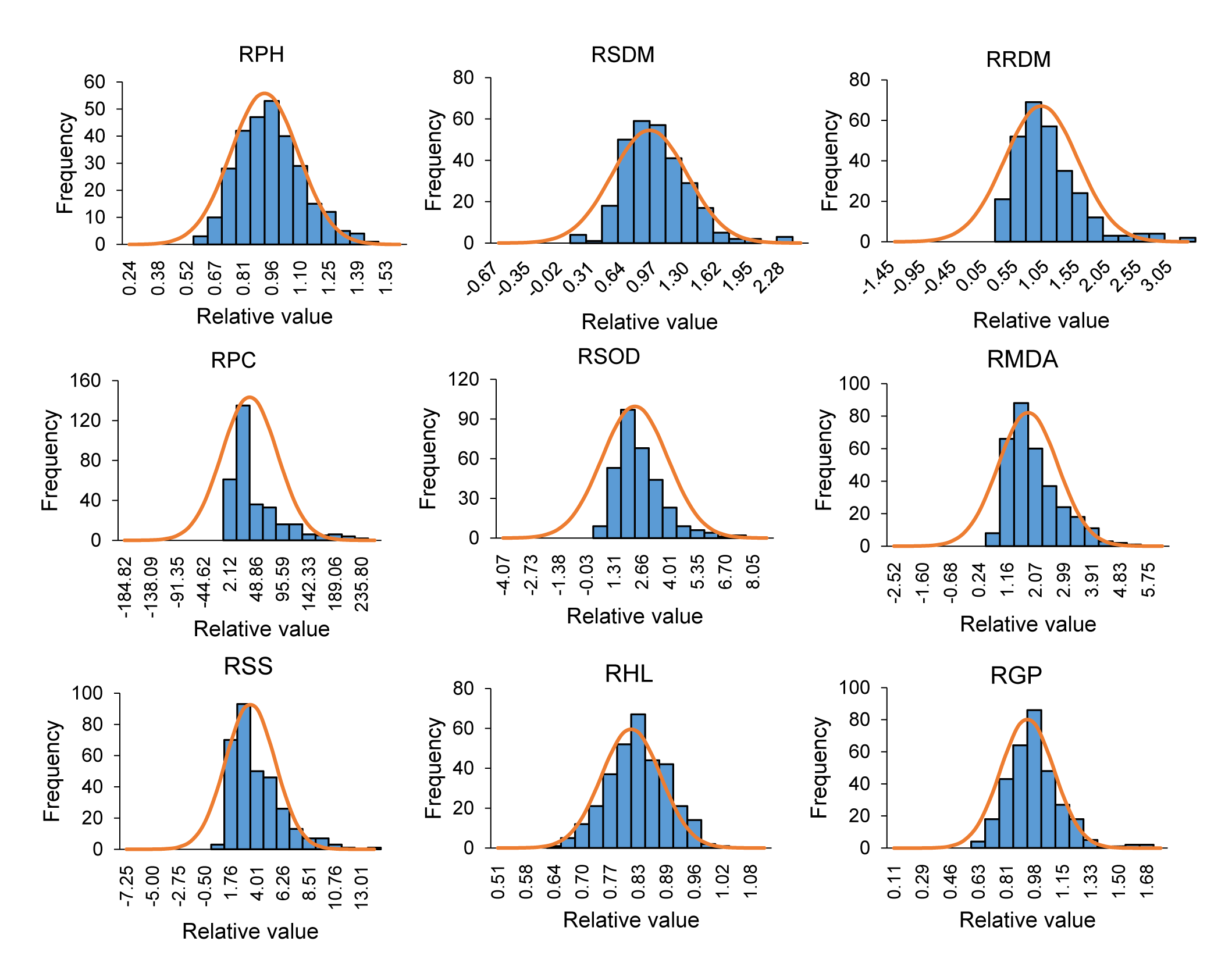

Supplement: FIGURE S1 — Phenotypic distributions for drought-tolerance traits. RPH, relative plant height; RSDM, relative shoot dry matter; RRDM, relative root dry matter; RPC, relative proline content; RSOD, relative superoxide dismutase activity; RMDA, relative malonaldehyde content; RSS, relative soluble sugar content; RHL, relative hypocotyl length; RGP, relative germination percentage. Gaussian curve is shown in orange line for each trait. [file Image_1.tif]

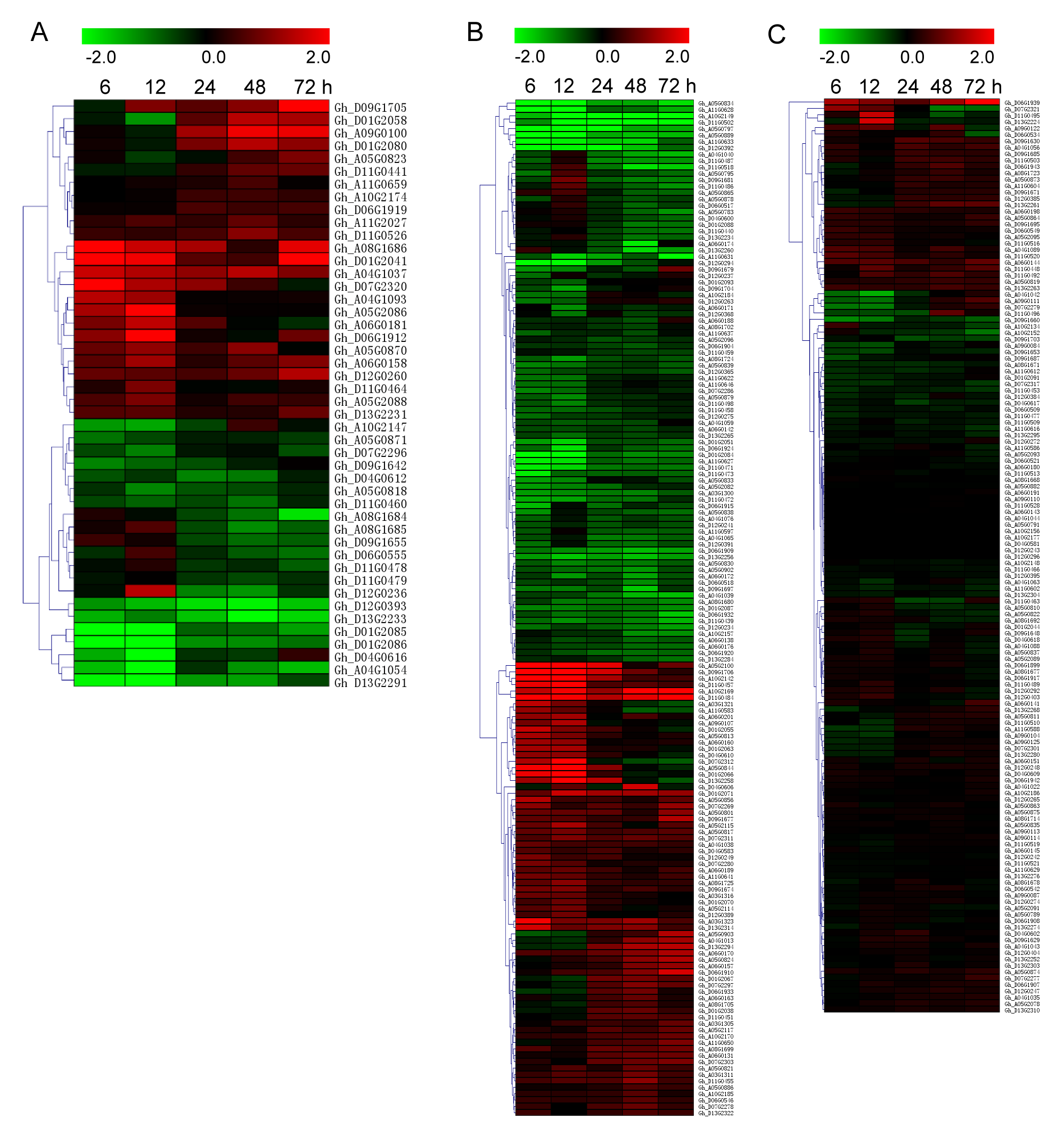

Supplement: FIGURE S2 — Expression pattern of the candidate genes involved in drought response. (A) Genes were annotated as stress response by Go analysis and also differential expression under drought stress. (B) Genes were not annotated as stress response by Go analysis but differential expression under drought stress. (C) Genes were annotated as stress response but not induced expression under drought stress. The scales represent the fold change value of gene expression level. [file Image_2.tif]
